# Correlation Between A Disintegrin and Metalloproteinase (ADAM) Family Proteins (8, 10, 17, 22) and Link with Neuroplasticity in Autism Spectrum Disorder

**DOI:** 10.3390/cimb47120980

**Published:** 2025-11-25

**Authors:** Dost Muhammad Halepoto, Laila Al-Ayadhi, Abdulrahman Alhowikan, Nadra Elamin, Durria Abdulmaged, Aurangzeb Halepota, Sarah Al-Mazidi

**Affiliations:** 1 Autism Research and Treatment Center, Al-Amodi Autism Research Chair, Department of Physiology, Faculty of Medicine, King Saud University, P.O. Box 2925, Riyadh 11461, Saudi Arabia; ayadh2@gmail.com (L.A.-A.); nadraelyass@hotmail.com (N.E.); duahmed@ksu.edu.sa (D.A.); 2 Department of Physiology, Faculty of Medicine, King Saud University, P.O. Box 2925, Riyadh 11461, Saudi Arabia; ahowikan@ksu.edu.sa (A.A.); athalepota@yahoo.com (A.H.); 3 Department of Anatomy and Physiology, College of Medicine, Imam Mohammad Ibn Saud Islamic University, P.O. Box 5701, Riyadh 11432, Saudi Arabia; s.almazeedi@gmail.com

**Keywords:** autism spectrum disorder, A disintegrin and metalloproteinase, neuroplasticity

## Abstract

**Background:** Autism spectrum disorder (ASD) is a condition related to neurodevelopment, typically identified by impaired social interactions and repetitive stereotypical behaviors. The etiology of ASD is not well known, but neuroplasticity has been suggested to play a pathological role. A disintegrin and metalloproteinases (ADAMs) are multifunctional transmembrane proteins that are important for development, communication, and plasticity in the nervous system. This study aimed to determine the correlations among ADAM proteins (ADAM-8, 10, 17, and 22) in children with ASD and to discuss their potential roles as molecular contributors to processes underlying neuroplasticity. **Methods:** The Spearman correlation coefficient (r) between plasma levels of ADAM 8, 10, 17, and 22 in children with ASD (n = 40) was obtained using the Statistical Package for the Social Sciences software, SPSS. **Results:** A significant correlation was obtained between plasma levels of the ADAM 8 and ADAM-17 (r = 0.523, *p ≤* 0.001); ADAM 8 and ADAM 22 (r = 0.576, *p* ≤ 0.001); and ADAM 17 and ADAM 22 (r = 0.489, *p* ≤ 0.001). However, no significant correlation between ADAM 10 and ADAM 8 (r = −0.147, *p* = 0.372); ADAM-17 (r = −0.143, *p* = 0.378), and ADAM-22 (r = −0.066, *p* = 0.684), was obtained. **Conclusions:** This study provides the first evidence of associations among circulating ADAM proteins (ADAM-8, ADAM-17, and ADAM-22) in children with ASD, suggesting their potential molecular involvement in pathways related to neuroplasticity. Further studies with larger cohorts and direct neuroplasticity measures are needed to clarify these relationships and their relevance to ASD pathophysiology.

## 1. Introduction

Autism spectrum disorder (ASD) is a complex neurodevelopmental condition typically identified by impaired social interactions and repetitive stereotypical behaviors [1]. The etiology of ASD is multifactorial, linked to many pathologies, including autoimmunity, neuroinflammation, genetic and other brain development abnormalities [2,3]. Neuroplasticity refers to the structural and functional variations that happen in the central nervous system (CNS) to adapt and respond to changes in the external environment; subjects with ASD show abnormal neuroplasticity, which affects information and sensory processing with social cognition, resulting in the manifestation of conforming symptoms. Gathering evidence suggests the significance of altered neuroplasticity in the CNS. However, understanding the mechanism that controls neuroplasticity in ASD is very important for identifying possible therapeutic targets [4].

A disintegrin and metalloproteinases (ADAMs) are multifunctional transmembrane proteins that are important for development, communication, and plasticity in the nervous system [5]. ADAMs are highly expressed in the nervous system, playing vital roles in cell adhesion, proteolysis, and signaling pathways within the CNS [6]. The functions of ADAMs in the nervous system and their use as drug targets for neurological and psychiatric diseases are discussed [7]. ADAMs also play a crucial role in reproduction and fertilization [8] and regulate various proteins, including adhesion molecules, cytokines, receptors, and growth factors, which are essential for different biological processes such as cell adhesion, cell migration, proteolysis, and signal transmission [9].

About thirty (30) ADAM family members are identified in the CNS, including ADAM8, ADAM10, ADAM17, and ADAM22, which have been linked with synaptic formation, remodeling, and neuronal communication [7]. Regardless of their recognized functions in neural development, the association between these proteins and the abnormal neuroplasticity observed in ASD remains poorly understood.

### 1.1. A Disintegrin and Metalloproteinase Protein 8 (ADAM 8)

A disintegrin and metalloproteinase 8 (ADAM8) is expressed in the central nervous system (CNS), mainly in immune system cells, dendritic cells, eosinophils, neutrophils, and monocytes [10]. ADAM8 has been implicated in several biological processes, such as muscle growth, neurogenesis, and connections between cells and matrices. ADAM8’s individual expression structure suggested potential functions in immunology and neuropathology [11]. A disintegrin and metalloproteinase 8 (ADAM8) is essential for inflammation and tumor-related diseases and has been found in numerous pathological conditions [12,13]. Furthermore, earlier research has shown that ADAM8 is important for both neuroinflammation and allergies [10,12]. Nonetheless, the molecular mechanism of ADAM8 is still unclear.

Very little research has been published to date regarding the ADAM8 protein’s involvement in ASD. Our group recently measured [11] that children with ASD had significantly lower plasma levels of ADAM8 than healthy controls; however, the severity of ASD does not seem to be directly correlated with these changes. These findings point to a potentially compromised anti-inflammatory mechanism that could influence ASD neurodevelopmental pathways.

### 1.2. A Disintegrin and Metalloproteinase Protein 10 (ADAM 10)

A Disintegrin and Metalloproteases 10 (ADAM10) is a multifunctional protein, highly expressed in the brain [14]. It controls brain development, synaptic functions, neuroinflammation, and immunity, which are involved in the pathogenesis of ASD [15,16]. ADAM10 has been explored more as a critical molecule in synapse formation and plasticity [17]. A recent study demonstrates how higher expression levels of ADAM10 can induce synaptic dysfunction in the brain by processing its substrates located in the synapse, and ultimately, processing can affect their function [18,19]. Although extensive research has examined the role of ADAM10 in neurological disorders, very little attention has been focused on ADAM10 in ASD. Studies have implicated changes in the plasma level of ADAM10 in the cognitive dysfunction of an older population [20,21]. A recent study demonstrated that activity and lower levels of ADAM10 caused abnormalities in the innate immune response of microglial cells, resulting in deficits in social behavior and repetitive behaviors in mice [16]. Recently, Al-Mazidi et al. [22] reported lower ADAM10 plasma levels and their relationship with cognition in children with ASD compared with normal children of the same age.

Previous investigations proposed an association of ADAM10 with social interaction impairment, learning deficiency, and other characteristic features that are present in ASD subjects [23,24]. However, additional evidence is needed to conclude whether ADAM10 is implicated in ASD.

### 1.3. A Disintegrin and Metalloproteinase Protein 17 (ADAM 17)

ADAM17 is a crucial regulator of numerous proteins implicated in the pathophysiology of ASD, including those involved in synaptic formation, axon signaling, neuroinflammation, brain development, and immunity [15,16]. ADAM17 is expressed in the human brain, and its expression in the fetal brain is significantly higher than in the adult brain [25]. This suggests that ADAM17 plays important roles in brain development [26]. Important protein substrates, including the neuro-inflammatory cytokine Tumor Necrosis Factor (TNF-α) and synaptic molecules Neural Glial-Related Cell Adhesion Molecules (NrCAM) that control neuronal networks and immune responses, respectively, are cleaved by ADAM17, both are implicated in or disrupted in ASD [15]. Zheng et al. [16] investigated the relationship between *p*-cresyl sulfate (*p*CS) and ADAM17, in the disrupted immune response of microglial cells in vitro linked to the pathophysiology of ASD.

ADAM17 plays a crucial role in numerous biological processes, such as development, immunological responses, cell proliferation, and survival [27]. Another role for ADAM17 is neural plasticity, or the brain’s capacity to alter its neural connections over the course of a lifetime.

The link between ADAM17 and ASD has been the subject of very little research, and its role in the pathophysiology of ASD is still unknown. Ray et al. [28] found a significantly higher level of soluble ADAM17 in the brain tissue of ASD patients. However, Al-Mazidi et al. [29] recently found that children with ASD had substantially lower plasma levels of ADAM17 than controls.

### 1.4. A Disintegrin and Metalloproteinase Protein 22 (ADAM 22)

ADAM22 cleaves important protein substrates that regulate immunological responses and neuronal networks, including glutamate synaptic molecules and the neuro-inflammatory cytokine TNF-α, which are implicated in or disrupted in ASD [30]. The ADAM22 protein is also involved in synaptic plasticity and function, both of which are essential for normal brain growth and function, and abnormalities in these processes are characteristic of ASD [29].

ADAM22 is highly expressed in the human brain [31] with the most prominent expression in cerebellum (granule cells) and hippocampus (CA1 pyramidal neurons). It is mainly involved in direct cell–cell communication by interacting with other proteins through their disintegrin domain [32].

Research has shown that changes in ADAM22 proteins relating to neuroinflammation and synapse functions as a possible underlying cause for the development of ASD. Moreover, lower plasma levels of ADAM22 have been observed in children with ASD [29]. Research also suggests that ADAM22 may be involved in glutamatergic signaling and synaptic transmission and may be dysregulated in individuals with ASD [29].

This study aimed to determine the correlations among ADAM proteins (ADAM-8, 10, 17, and 22) in children with ASD and to discuss their potential roles as molecular contributors to processes underlying neuroplasticity.

## 2. Materials and Methods

### 2.1. Participants

The study was carried out at the Autism Research and Treatment Center (ARTC), Faculty of Medicine, King Saud University, and King Khalid University Hospital. This study enrolled eighty children (aged 3–13 years), including 40 children with ASD from the ARTC and 40 healthy children from the pediatric clinic at King Khalid University Hospital, based on the inclusion criteria and protocol. The diagnosis of ASD was made according to the 5th edition of the Diagnostic and Statistical Manual of Mental Disorders (DSM–5) [1]. Participants with obsessive–compulsive disorder, affective disorders, fragile X syndrome, epileptic seizures, or any other neurological or psychiatric conditions were excluded from the study.

The study was approved by the Institutional Review Board of the Faculty of Medicine, King Saud University. A signed informed written consent was obtained from the parents or the legal guardians of children with ASD before enrolling in the study. All procedures followed the Helsinki Declaration for human investigations. The plasma ADAM protein data used in this study were derived from previously collected datasets published by our group; however, the present analysis represents a new investigation focusing on the correlation of ADAM proteins with neuroplasticity markers, which has not been addressed in prior reports.

### 2.2. Behavioral Assessment

The severity of ASD, as well as social and sensory performances, were assessed in all children with ASD using the Childhood Autism Rating Scale (CARS), Short Sensory Profile (SSP), and Social Responsiveness Scale (SRS) as reported previously by our research groups [11,22,29].

### 2.3. Blood Sample Collection and Measurement of Plasma Levels by ELISA

Blood plasma levels of all ADAMs were measured as reported previously [11,22,29] using a commercially available sandwich Enzyme-Linked Immunosorbent Assay (ELISA) kit (Cusabio Biotech Co., Ltd., Wuhan, China).

### 2.4. Statistical Analysis

The Statistical Package for the Social Sciences (SPSS) version 26 (IBM Corp., Chicago, IL, USA) was used to analyze the data. Normality of data was tested by the Shapiro–Wilk test. The findings were displayed as mean ± standard deviation. All statistical comparisons were conducted using Independent Student’s *t*-tests, where a *p*-value of less than 0.05 is deemed significant. The correlation between the ADAM 8, ADAM 10, ADAM 17, and ADAM 22 was determined using the Spearman correlation (r) test.

## 3. Results

**Figure 1:**
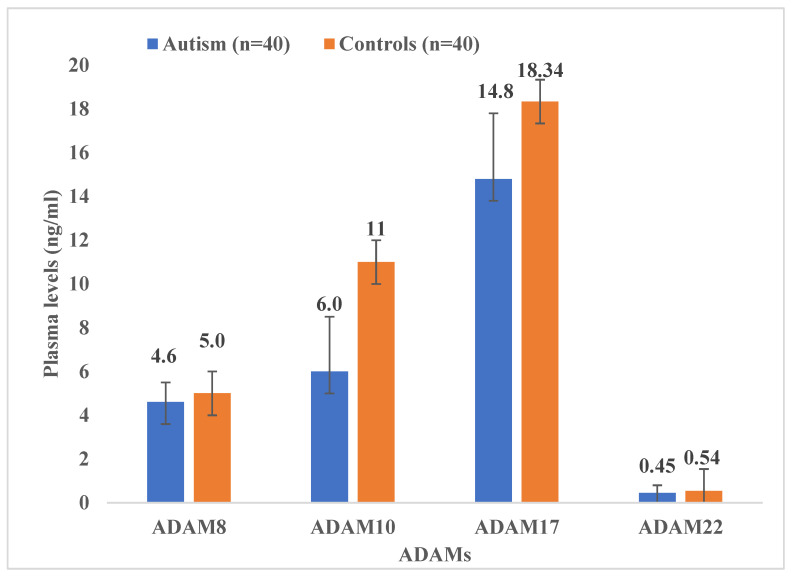
Plasma levels of ADAMs in children with ASD and healthy controls. ADAM 8 (*p* ≤ 0.05); ADAM 10 (*p* < 0.05); ADAM 17 (*p* = 0.006); ADAM 22 (*p* = 0.41).

**Figure 2:**
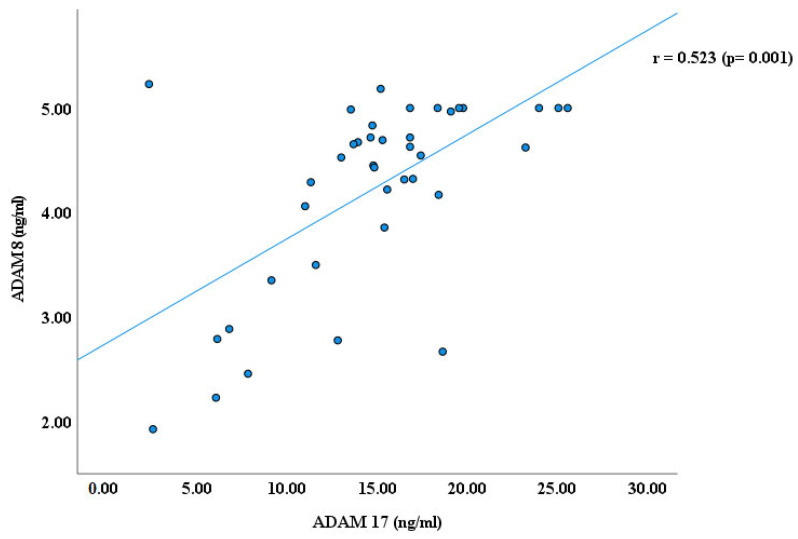
Spearman’s Correlation (r) between ADAM 8 and ADAM 17.

**Figure 3:**
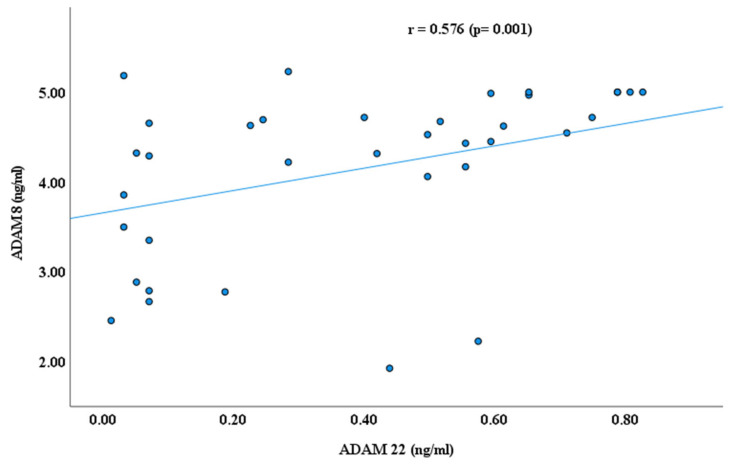
Spearman’s Correlation (r) between ADAM 8 and ADAM 22.

**Figure 4:**
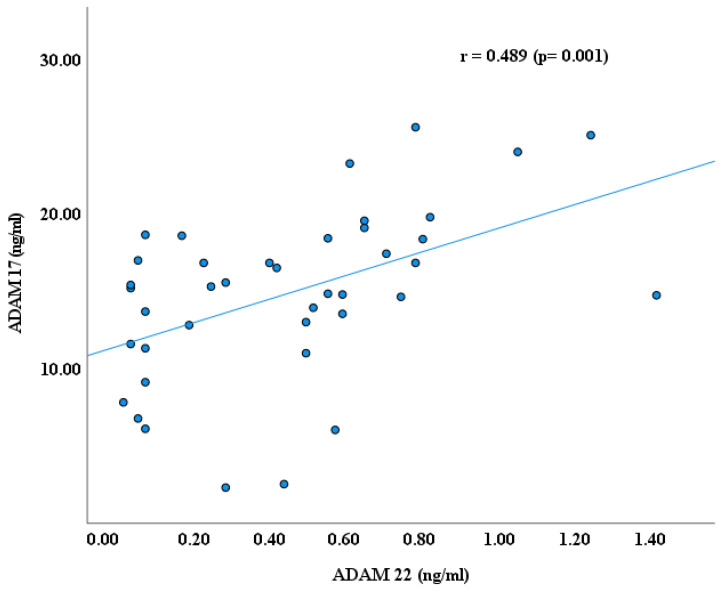
Spearman’s Correlation (r) between ADAM 17 and ADAM 22.

**Figure 5:**
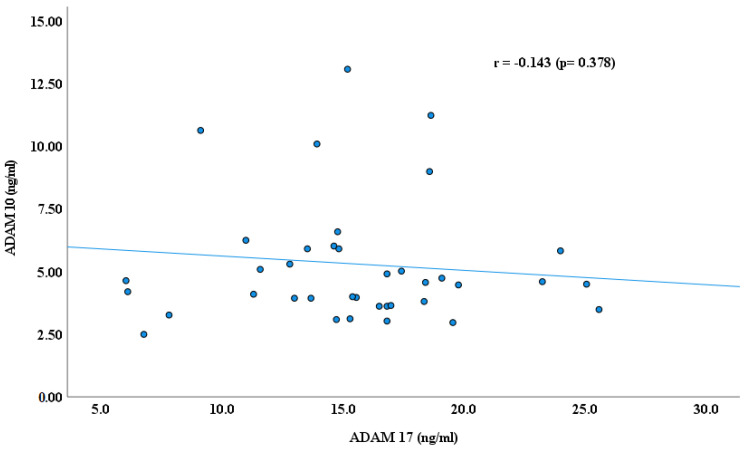
Spearman’s Correlation (r) between ADAM 10 and ADAM 17.

**Figure 6:**
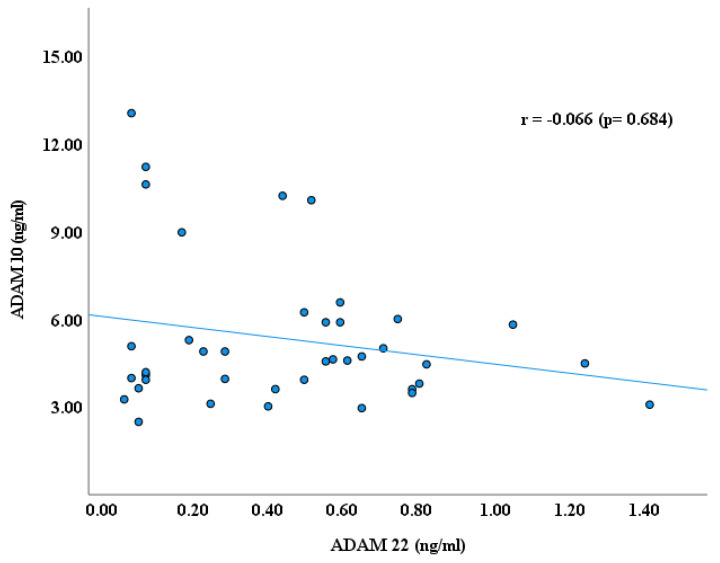
Spearman’s Correlation (r) between ADAM 10 and ADAM 22.

**Figure 7:**
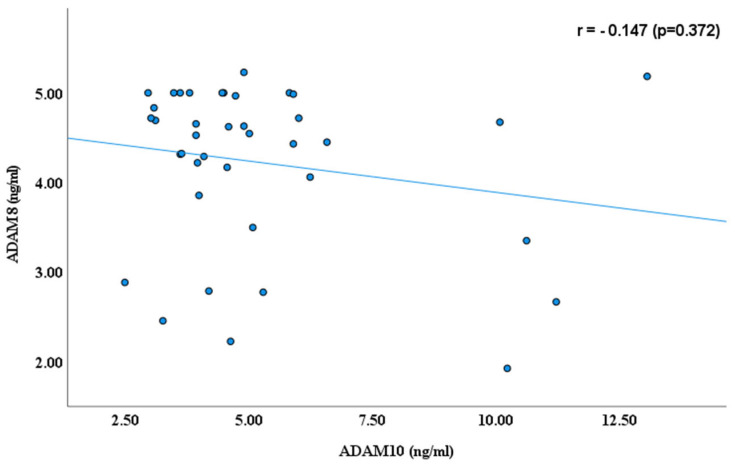
Spearman’s Correlation (r) between ADAM 8 and ADAM 10.

As reported earlier by our research groups [11,22,29], the blood plasma levels of ADAM proteins, including ADAM 8, 10,17, and 22, were significantly lower in autistic children than in healthy controls. ADAM 17 has higher plasma values than ADAMs 8, 10, and ADAM 22 in children with ASD, as shown in Figure 1. In continuity of these results, significant Spearman’s correlation coefficients (r) were obtained between ADAM-8 and ADAM-17 (r = 0.523, *p* ≤ 0.001); ADAM 8 and ADAM 22 (r = 0.576, *p* ≤ 0.001); and ADAM 17 and ADAM 22 (r = 0.489, *p* ≤ 0.001), as shown in Figure 2, Figure 3 and Figure 4. However, no significant correlation between ADAM 10 and ADAM 8 (r = −0.147, *p* = 0.372), ADAM-17 (r = −0.143, *p* = 0.378), and ADAM-22 (r = −0.066, *p* = 0.684) was observed, as shown in Figure 5, Figure 6 and Figure 7.

## 4. Discussion

Autism spectrum disorder (ASD) is a neurodevelopmental disorder characterized by various etiologies and mechanisms involved in abnormal brain development [22]. Increasing evidence suggests that neuroinflammatory processes play a critical role in ASD, and ADAM family proteins may contribute to this mechanism [11,22]. However, the neurological basis of ASD is not clear, and the contribution of ADAMs needs to be examined.

The nervous system acts as the regulatory center of an organism that detects, interprets, and responds to outer or inner changes. These processes involve both intercellular communication and intra- and intercellular signaling events that rely on proteins expressed on the surface of cells to act at the lines of action [7].

ADAMs are multifunctional transmembrane proteases involved in intercellular signaling, adhesion, and proteolytic shedding of cell-surface proteins [6]. Several members of this family, particularly ADAM8 and ADAM17, are linked to immune activation and inflammatory regulation within the nervous system. ADAMs exhibit a wide range of expression patterns in many cell and tissue types and are involved in many biological processes, including synaptic formation and plasticity [6]. Overall, converging evidence from multiple areas of research on ASD suggested the implication of ADAMs in widespread abnormalities in brain development and functions in ASD [11,22,29]. Moreover, the integrated proteolytic network linking neuroinflammatory and synaptic processes in ASD.

Although ADAMs mainly function in the central nervous system, their soluble or shed forms can be detected in the peripheral circulation, which reflects both systemic and neuroimmune activity [7]. This means that plasma ADAM levels could serve as accessible peripheral biomarkers that, at least in part, mirror central processes like neuroinflammation, synaptic remodeling, and neuroplasticity. Previous studies have indicated that several members of the ADAM family—like ADAM10 and ADAM17—are found in soluble forms in blood and cerebrospinal fluid, playing roles in both central and peripheral signaling [33,34]. Additionally, changes in peripheral ADAM levels have been linked to neurological conditions, supporting their potential as biomarkers that connect peripheral and brain pathophysiology [33,35].

In the present study, a possible link was hypothesized among ADAMs 8, 10, 17, and 22, which may help in understanding the involvement of the ADAMs in ASD. Therefore, the goal of the present study was to investigate the relationships among ADAMs and their involvement with neuroplasticity in children with ASD.

According to our earlier research, children with ASD have substantially lower plasma levels of the ADAM family members (ADAM8, ADAM10, ADAM17, and ADAM22) than normal controls [11,22,29]. This decrease indicates a shared dysregulation at the molecular level affecting multiple proteases, which may be related to ASD’s compromised neuroplasticity, synaptic remodeling, and neuroimmune signaling. However, when we compare ADAM plasma levels with each other, ADAM17 showed comparatively higher plasma concentrations than ADAM8, ADAM10, and ADAM22 in ASD subjects (Figure 1). This suggests that it plays a key role in neurotrophic and inflammatory signaling as a major sheddase. Despite this relative elevation, ADAM17 levels were still much lower than those of healthy controls, indicating that ASD may affect this crucial α-secretase pathway. Decreased ADAM17 activity may hinder the processing of cytokines (e.g., TNF-α, IL-1β, and TGF-α) and attenuate the cleavage of amyloid precursor protein (APP) into neuroprotective sAPPα, resulting in deficiencies in immune modulation and synaptic maturation.

The plasma ADAM protein data used in this study have been described in our previous publications [11,22,29]; that work focused primarily on measuring plasma levels and their correlation with severity levels (CARS, SRS, SSP) in autistic children. However, the current study introduces a new analytical perspective by investigating the interrelationships among circulating levels of ADAM-8, ADAM-10, ADAM-17, and ADAM-22 in children with ASD and discussing their potential roles as molecular contributors to processes underlying neuroplasticity in ASD. Furthermore, this integrative correlation approach provides novel insights into the significant role of ADAM family proteins in the neurobiological framework of ASD.

Results of this study demonstrate significant correlations between ADAM8, ADAM17, and ADAM22 (*p* ≤ 0.001), suggesting potential molecular associations among specific ADAM proteins that may contribute to pathways involved in neuronal development relevant to ASD. However, the precise mechanisms through which ADAM proteins influence ASD pathophysiology remain unclear.

ADAM8, which is primarily associated with microglial activation and neuroinflammation [11], may influence ADAM22-mediated synaptic adhesion and ADAM17-dependent cytokine shedding [29]. This cross-regulation may lead to unified control of neuroplasticity, integrating immune activity with structural synaptic remodeling, which may aid in the integrated control of neuroplasticity. Such cross-regulation could contribute to integrated control of neuroplasticity, balancing immune reactivity with structural synaptic remodeling. The strong interdependence supports the idea that changes in a single ADAM member may cause dysregulation across a larger proteolytic network, intensifying neurodevelopmental irregularities in ASD.

On the other hand, there was no significant correlation for ADAM10 with any of the other ADAMs, implying that its role in ASD may be quite different or similarly independently regulated. ADAM10 is the main α-secretase for amyloid precursor protein (APP) processing; reduced activity may potentially affect the formation of sAPPα, a neurotrophic factor that is critical for dendritic growth and synaptic stabilization [22]. The lack of association with ADAM8, ADAM17, or ADAM22 suggests that ADAM10-related pathways may be differently impacted in ASD, possibly due to selective deficiencies in synaptic pruning or adhesion rather than widespread immuno-synaptic interactions. This finding supports previous reports that suggested that ADAM10-mediated CNTNAP2 processing, a gene implicated in ASD, disrupts neuronal adhesion and network formation after dysregulation [22].

Overall, these results support the idea that decreased systemic availability of ADAM proteins may affect both neuroimmune homeostasis and synaptic remodeling in ASD. The intercorrelations among ADAM8, ADAM17, and ADAM22 propose a functional axis where inflammatory regulation, cytokine processing, and synaptic adhesion converge [11,29]. The independence of ADAM10 may specify a more particular involvement in synaptic maturation that is, however, equally disrupted.

To our knowledge, this is the first study to report the correlation among ADAMs and their association with neuroplasticity in ASD. The findings need to be confirmed with a larger sample size to confirm these associations, which show promising biomarkers for early diagnosis and a possible therapeutic target for ASD.

This study has several limitations. The main limitation of this study is the relatively small sample size, which may limit the statistical power and generalizability of the findings. Moreover, the cross-sectional design makes it tough to conclude on the timing or cause-and-effect relationships between ADAM protein levels and processes related to ASD. Another significant limitation is the lack of direct assessments of neuroplasticity, like neuroimaging, electrophysiological measures, or analyses of neurotrophic factors, which would help validate the molecular correlations we have observed. Future research should focus on combining molecular tests of ADAM proteins with neuroimaging and behavioral evaluations to better understand their potential roles in neural plasticity and the pathophysiology of ASD.

## 5. Conclusions

ADAMs are potential novel biomarkers that assess the degree of cognitive, social, and sensory impairments related to ASD, which may correlate with the severity of the disorder. The significant associations of ADAMs (8, 17, and 22) may reflect a relationship to neural development in the brain, indicating their involvement in the early stages of the pathophysiology of ASD, where synaptic dysfunction is either directly or indirectly implicated. Therefore, there is a pressing need to characterize the preferential substrates of ADAMs burgeoning during different stages of the development of ASD. Future studies are strongly encouraged to investigate the critical contribution of ADAMs to the ASD phenotypes. Understanding of the relevant molecular mechanism may help in the development of a new therapeutic strategy for neuronal protection in ASD.

## Data Availability

The data presented in this study are available on request from the corresponding author if required (due to ethical reasons).

## Abbreviations

The following abbreviations are used in this manuscript:

ASD	Autism Spectrum Disorder
DSM–5	Diagnostic and Statistical Manual of Mental Disorders–5
ADAM	A disintegrin and metalloproteinase
ADAMs	A disintegrin and metalloproteinases
CNS	Central Nervous System
KACST	King Abdulaziz City for Science and Technology
APA	American Psychiatric Association
ARTC	Autism Research and Treatment Center
TNF-α	Tumor Necrosis Factor Alpha
NrCAM	Neural Glial-Related Cell Adhesion Molecules
*p*CS	*p*-cresyl sulfate
CARS	Childhood Autism Rating Scale
SRS	Social Responsiveness Scale
ELISA	Enzyme-Linked Immunosorbent Assay
SPSS	Statistical Package for the Social Sciences
APP	Amyloid precursor protein
IL-1β	Interleukin-1 beta
TGF-α	Transforming Growth Factor Alpha
sAPPα	Soluble Amyloid Precursor Protein Alpha
CNTNAP2	Contactin-associated protein-like 2
